# A case of a diverticulum-like giant jejunal gastrointestinal stromal tumour presenting with intraperitoneal peritonitis due to rupture

**DOI:** 10.1016/j.ijscr.2020.03.017

**Published:** 2020-04-01

**Authors:** Ryosuke Arata, Hideki Nakahara, Takashi Urushihara, Toshiyuki Itamoto, Takashi Nishisaka

**Affiliations:** a Department of Gastroenterological Surgery, Hiroshima Prefectural Hospital, Hiroshima, Japan; b Department of Gastroenterological and Transplant Surgery Applied Life Sciences, Institute of Biomedical and Health Sciences, Hiroshima University, Hiroshima, Japan; c Department of Pathology Clinical Laboratory, Hiroshima Prefectural Hospital, Hiroshima, Japan

**Keywords:** GIST, gastrointestinal stromal tumour, GIT, gastrointestinal tract, CT, computed tomography, Gastrointestinal stromal tumour, Small intestine, Rupture of the tumour, Peritonitis, Local resection, Chemotherapy

## Abstract

**Introduction:**

Early diagnosis of small intestinal gastrointestinal stromal tumours (GISTs) is difficult. These tumours often present with peritonitis and intraperitoneal bleeding due to rupture. We experienced a case of a tumor that grows to 12 cm asymptomatic.

**Presentation of case:**

A 46-year-old man presented with sudden abdominal pain. Computed tomography revealed free air in the abdominal cavity, a 12-cm sized dilatation in the small intestine, and pooled residues. He was diagnosed with perforation of the gastrointestinal tract, and emergency surgery was performed. Inside the abdominal cavity, approximately 500 mL of bloody ascites was observed. A diverticulum-like mass measuring approximately 12-cm long was observed on the jejunum approximately 30 cm from the Treitz ligament. There was a large hematoma inside the mass, and perforation was recognised at the neck of the diverticulum. Partial resection of the jejunum and intraperitoneal drainage were performed. Immunohistochemistry revealed that the tumour was positive for KIT and CD34, and GIST was diagnosed. The patient was discharged on postoperative day 10 without significant complications. The patient did not relapse while taking imatinib as an adjuvant chemotherapy.

**Discussion:**

Gastrointestinal stromal tumours are the most common mesenchymal tumours of the gastrointestinal tract and may undergo increased diverticulum-like growth, as seen in this case. Local resection and proper chemotherapy increase long-term survival, suppress tumour growth, and reduce the risk of relapse.

**Conclusion:**

Diagnosing GIST can be difficult due to the absence of clinical symptoms. It is necessary to ensure local resection and careful long-term follow-up.

## Highlights

• Diagnosing GIST is difficult due to the absence of clinical symptoms.
• GIST should be considered when sudden abdominal pain and a mass are present.
• We present a case of a GIST with partial resection and drainage surgery.
• Ensuring local resection and proper chemotherapy increases long-term survival.

## 1 Introduction

Gastrointestinal stromal tumours (GISTs) are the most common mesenchymal tumours of the gastrointestinal tract (GIT) [1]. Approximately 60–70% of these tumours occur in the stomach, 20–30% occur in the small intestine, and 5% occur in other areas of the GIT [2,3]. Stomach GISTs are frequently diagnosed in asymptomatic patients because there are many opportunities for direct observation during health examinations [4]. However, small intestinal GISTs are often found when symptoms appear; hence, the size of intestinal GISTs at diagnosis is typically large. Approximately two-thirds of GISTs in the small intestine are 5 cm or more in diameter at the time of diagnosis and are rarely 2 cm or less [5]. The clinical symptoms of GISTs range from mild to severe, and complications include vague abdominal pain, hematemesis, and intestinal obstruction. Although obvious peritonitis due to the rupture of a GIST is relatively rare [6], considering spontaneous rupture of small intestinal GISTs is important in order to perform radical resection during emergency surgery. We report a case of a diverticulum-like small intestinal GIST in an asymptomatic patient. The GIST had grown to a size of 12 cm long and had ruptured.

This work has been reported in line with the SCARE criteria [7].

## 2 Presentation of case

**Fig. 1:**
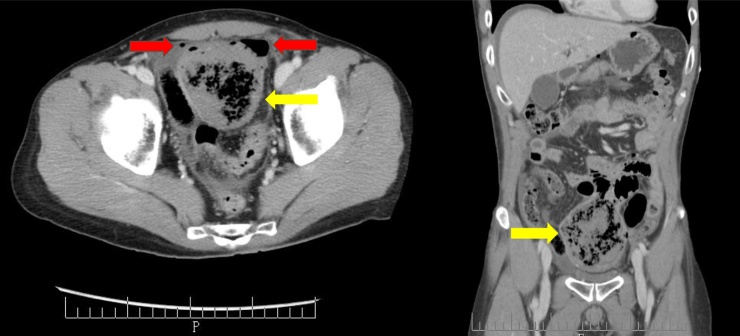
Computed tomography findings. A 12-cm cystic dilatation is observed in the small intestine, and pooled residues are seen inside the abdominal cavity (yellow arrows). Free air is found in the abdominal cavity (red arrows).

**Fig. 2:**
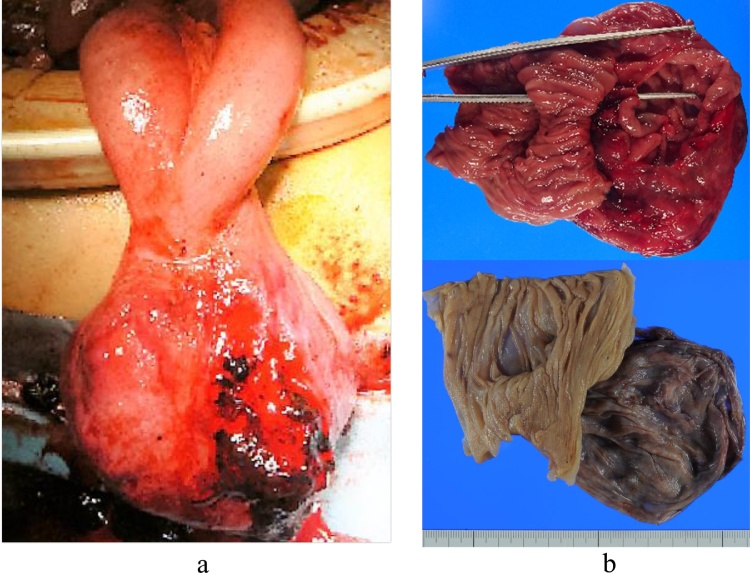
Intraoperative and postoperative findings. (a) Intraoperative photograph showing a large tumour (measuring 10 cm) with extraluminal growth arising from the jejunum. The tumour was ligated 30 cm from the Treitz ligament, and there was a large hematoma inside. Perforation was recognised at the neck of the same site. (b) Postoperative photograph showing the excised specimen. The tumour is 7.0 × 6.5 cm in size and is comprised mostly of cystic lesions containing blood. It shows extraluminal development on the mesenteric side, and the tumour penetrates the mucosal surface.

A 46-year-old man who was suffering from severe abdominal pain was brought to our hospital by ambulance. Examination revealed abdominal tenderness and guarding in the upper abdomen. Computed tomography (CT) showed free air in the abdominal cavity, a 12-cm cystic dilatation in the small intestine, and pooled residues inside the abdomen (Fig. 1). Because these findings suggested peritonitis induced by perforation of the upper GIT, we performed emergency surgery. About 500 mL of bloody ascites was observed in the abdominal cavity. A diverticulum, approximately 12 cm in size, was observed on the jejunum. There was a large hematoma within the mass, and perforation was recognised at the neck of the diverticulum (Fig. 2a). We suspected perforation of a giant diverticulum in the jejunum; thus, partial resection of the jejunum and intraperitoneal drainage were performed.

**Fig. 3:**
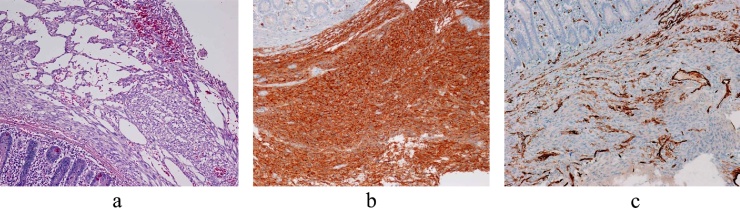
Immunochemistry findings. (a) Haematoxylin and eosin staining showing proliferation of spindle-shaped cells. (b) and (c) Immunohistochemistry reveals that the tumour is positive for KIT (b) and　CD34 (c).

The excised specimen revealed that the mass-like diverticulum was 7.0 × 6.5 cm in size and contained blood. It showed that the mass had developed on the antimesenteric side of the jejunum, and it connected with the lumen of the jejunum (Fig. 2b). Haematoxylin and eosin staining showed proliferation of spindle-shaped cells, and immunohistochemical staining revealed that the tumour was positive for KIT and CD34, with approximately 4.0% of the tumour cells positive for nuclear expression of the proliferation-associated antigen Ki-67 (Fig. 3). Therefore, the patient was diagnosed with a high-risk GIST of the jejunum. The patient was discharged on postoperative day 10 without complications. When the diagnosis was confirmed by histology, the patient immediately received imatinib mesylate therapy. He is currently under follow-up without recurrence or peritoneal dissemination.

## 3 Discussion

Diagnosis of small intestine GISTs is difficult, and it is often incidentally made during surgery, endoscopy, radiology, or at the time of autopsy [9,10]. Common gross patterns have been classified into four types: endoluminal, exoluminal, intramural, and mixed (hourglass or dumb-bell shaped) [11]. There were no GIST cells in the lumen of the small intestine, no mucosal structure was found in the diverticulated part, and GIST cells were found in the luminal aspect of the diverticulated part. Furthermore, there was no evidence of ectopic gastric mucosa or pancreatic tissue and no evidence of GIST coexisting with Meckel’s diverticulum. Therefore, the GIST did not originate from the diverticulum that originally existed. It was thought to have first developed from an exoluminal growth in the jejunum, forming a pouch in the gastrointestinal lumen, which then formed a diverticulum. Ikemura et al. reported that although the mechanism underlying the diverticulum-like structure of the GIST has not been determined, this GIST, causing diverticulum-like structures of the gastrointestinal tract, should be recognised as a specific gut wall replacing type of GIST [12]. There are few reports of diverticulum-like growth, especially in cases where symptoms appear for the first time beyond 12 cm, as in this case. There has been no systematic analysis of whether the macroscopic growth pattern of the GIST is associated with prognosis. Although most of these small tumours are asymptomatic, GISTs ≥4 cm may produce symptoms secondary to obstruction or GI bleeding [8,13]. GISTs originating from the small bowel may cause perforation and are more frequently in the jejunum than in the ileum [13]. Spontaneously ruptured small GISTs vary from diminutive nodules to complex masses >20 cm that extend into the abdomen, and the tumours are generally considered to be ≥5 cm [5]; in this case, the tumour was approximately 12 cm. However, there have been reports of tumours rupturing at a size of 2 cm; therefore, if the size is large, the possibility of rupture increases, but there is a risk of rupture even if the size is small [14]. There is no consensus or universally accepted definition for ”tumour rupture”, and the reported incidence of tumour rupture varies greatly, from 1 to 27% [15]. Recently, Nishida et al. suggested the following six criteria for “tumour rupture”: (i) tumour fracture or spillage, (ii) blood-stained ascites, (iii) gastrointestinal perforation at the tumour site, (iv) microscopic infiltration of an adjacent organ, (v) intralesional dissection or piecemeal resection, or (vi) incisional biopsy [15]. As in this case, bowel perforation type ruptures may be caused by obstruction with increasing intraluminal pressure or an erosive tumour leading to mural necrosis and perforation [16]. The cells were separated and sparse near the perforation site. The increased size of the GIST caused changes from the inside due to ischemia or necrosis, and it was thought that the fragile wall had broken down, resulting in perforation. The GIST, causing diverticulum-like structures of the GIT, did not infiltrate the serosa and may clinically appear to be more like a GI perforation than a ruptured tumour. Therefore, this type of GIST may be less likely to result in peritoneal metastases due to rupture; however, some studies have demonstrated that recurrences after rupture were frequently peritoneal [17,18]. This risk of peritoneal recurrence after tumour rupture may be high, indicating that tumour rupture may be an important prognostic factor in GIST. Complete surgical resection is considered the only potential curative treatment for localised GISTs. Tumour rupture may result in peritoneal seeding of tumour cells; hence, surgery may be R1 surgery even if achieving a macroscopically complete resection. Complete resection can be achieved in approximately 85% of patients, and the estimated incidence of recurrence or metastasis after radical surgery is 50% [10]. Patient prognosis is poor when tumours are accompanied by symptoms or signs such as perforation or rupture, multifocal location, or metastatic lesions. Targeted therapy using imatinib, a first-generation moderately toxic tyrosine kinase inhibitor administered after surgery, increases survival and suppresses tumour growth [19]. In patients with severe adverse reactions to imatinib or those who show an insufficient response, second-generation tyrosine kinase inhibitors like sunitinib or regorafenib have also been reported to be effective [20]. In this case, when the postoperative diagnosis was made, imatinib was immediately administered. The patient continues to receive imatinib and is currently alive without evidence of recurrence.

## 4 Conclusion

This case reports a relatively rare case of an asymptomatic patient with a 12-cm diverticulum-like GIST in the jejunum that ruptured spontaneously. Preoperative diagnosis of small intestinal GISTs is often difficult because of the absence of clinical symptoms. Consequently, GISTs should always be considered in the differential diagnosis of peritonitis in patients with an intra-abdominal mass. If GIST rupture is suspected, it is important to perform radical resection, continue appropriate treatment after surgery, and closely follow the patient and observe for relapse.

## Declaration of Competing Interest

The authors have no conflicts of interest.

## Sources of funding

This research did not receive any specific grant from funding agencies in the public, commercial, or not-for-profit sectors.

## Ethical approval

Ethical approval was not required and patient identifying knowledge was not presented in the report.

## Consent

Written informed consent has been obtained from the patient for the publication of this case report and any accompanying images.

## Author contribution

RA and HN participated in treatment of the patient, collected case details, literature search and draft the manuscript. TU and TN participated in treatment planning of the patient. TI participated in treatment planning of the patient and helped to draft the manuscript. All authors read and approved the final manuscript.

## Registration of research studies

Not applicable.

## Guarantor

Hideki Nakahara.

## Provenance and peer review

Not commissioned, externally peer-reviewed.
